# Acclimation of C_4_ metabolism to low light in mature maize leaves could limit energetic losses during progressive shading in a crop canopy

**DOI:** 10.1093/jxb/eru052

**Published:** 2014-03-03

**Authors:** Chandra Bellasio, Howard Griffiths

**Affiliations:** Physiological Ecology Group, Department of Plant Sciences, University of Cambridge, Downing Street, Cambridge CB2 3EA, UK

**Keywords:** Bundle sheath, Δ ^13^C, irradiance, isotopic discrimination, leakiness, low light, mesophyll, efficiency, PPFD.

## Abstract

C_4_ plants have a biochemical carbon-concentrating mechanism that increases CO_2_ concentration around Rubisco in the bundle sheath. Under low light, the activity of the carbon-concentrating mechanism generally decreases, associated with an increase in leakiness (*ϕ*), the ratio of CO_2_ retrodiffusing from the bundle sheath relative to C_4_ carboxylation. This increase in *ϕ* had been theoretically associated with a decrease in biochemical operating efficiency (expressed as ATP cost of gross assimilation, ATP/*GA*) under low light and, because a proportion of canopy photosynthesis is carried out by shaded leaves, potential productivity losses at field scale. Maize plants were grown under light regimes representing the cycle that leaves undergo in the canopy, whereby younger leaves initially developed under high light and were then re-acclimated to low light (600 to 100 μE·m^−2^·s^−1^ photosynthetically active radiation) for 3 weeks. Following re-acclimation, leaves reduced rates of light-respiration and reached a status of lower *ϕ*, effectively optimizing the limited ATP resources available under low photosynthetically active radiation. Direct estimates of respiration in the light, and ATP production rate, allowed an empirical estimate of ATP production rate relative to gross assimilation to be derived. These values were compared to modelled ATP/*GA* which was predicted using leakiness as the sole proxy for ATP/*GA*, and, using a novel comprehensive biochemical model, showing that irrespective of whether leaves are acclimated to very low or high light intensity, the biochemical efficiency of the C_4_ cycle does not decrease at low photosynthetically active radiation.

## Introduction

The C_4_ pathway of photosynthesis has been attracting increasing interest in recent years for its high crop productivity potential in the face of global warming and population pressure (Friso *et al.*, 2010; Zhu *et al.*, 2010; Covshoff and Hibberd, 2012). C_4_ photosynthesis evolved from C_3_ photosynthesis under the environmental pressure of declining ambient CO_2_ and increasing transpiration demand in semi-arid environments (Griffiths *et al.*, 2013; Osborne and Sack, 2012). Under optimal conditions, characterized by high temperatures and high light intensities, C_4_ plants have higher photosynthetic rates than C_3_ plants (Ehleringer and Pearcy, 1983; Pearcy and Ehleringer, 1984) and very high productivity. Many C_4_ plants have been domesticated and represent irreplaceable sources of food, biomass, and bioenergy. For instance, maize (*Zea mays*, L.), a C_4_ plant of the NADP-malic enzyme (NADP-ME) subtype, is the leading grain production cereal (www.fao.org/statistics).

The high productivity of C_4_ plants results from anatomical and biochemical differentiation of the leaf parenchyma. Externally mesophyll cells and internally bundle sheath (BS) cells are coupled to operate a biochemical carbon-concentrating mechanism (CCM) that increases the CO_2_ concentration in BS, the cellular compartment where Rubisco is exclusively expressed, resulting in active suppression of the oxygenase activity of Rubisco. Since BS and mesophyll cells are connected by plasmodesmata, some CO_2_ retrodiffuses (CO_2_ leakage). The extent of CO_2_ retrodiffusion is still debated, but it is accepted that the permeability to CO_2_ diffusion (BS conductance, *g*_*BS*_; Table 1) varies between different species and individual plants. A useful term to describe this concept, which was coined by Farquhar in the description of carbon isotope discrimination (Farquhar, 1983) is leakiness (*ϕ*), defined as the rate of CO_2_ retrodiffusing (leak rate) relative to the phosphoenolpyruvate (PEP) carboxylation rate (*V*_*P*_). Since Rubisco CO_2_ fixation (in BS) is complementary to leakage (out of BS), *ϕ* can be used as a proxy for the coordination between the CCM and C_3_ assimilatory activity (Henderson *et al.*, 1992; von Caemmerer, 2000; Tazoe *et al.*, 2006, 2008; Kromdijk *et al.*, 2010; Ubierna *et al.*, 2011; Bellasio and Griffiths, 2013).

**Table 1.** Definitions, equations, and variables used

Symbol	Definition	Values/units/references
*A*	Net assimilation	μmol·m^−2^·s^−1^
*a*	^13^C fractionation due to diffusion of CO_2_ in air. Due to vigorous ventilation we ignored fractionation at the boundary layer.	4.4‰ (Craig, 1953; Kromdijk *et al.*, 2010)
*a*_*d*_	^13^C fractionation due to diffusion of CO_2_ in water	0.7‰ (O’Leary, 1984)
ATP/*GA*	Predicted ATP demand for gross assimilation, i.e. predicted biochemical operating efficiency	μmol·m^−2^·s^−1^
*b*_3_	^13^C fractionation during carboxylation by Rubisco including respiration and photorespiration fractionation $b_{3}=b_{3}\prime -\frac{e\prime \cdot R_{LIGHT}+f\cdot \text{ F}}{V_{c}}$	‰ (Farquhar, 1983; Ubierna *et al.*, 2013)
*b*_3_′	^13^C fractionation during carboxylation by Rubisco (excluding respiration and photorespiration fractionation)	30‰ (Roeske and Oleary, 1984)
*b*_4_	Net fractionation by CO_2_ dissolution, hydration and PEPC carboxylation including respiratory fractionation $b_{4}=b_{4}\prime -\frac{e\prime R_{M}}{V_{P}}$	‰ (Farquhar, 1983; Henderson *et al.*, 1992)
*b*_4_′	Net fractionation by CO_2_ dissolution, hydration, and PEPC carboxylation (excluding respiratory fractionation)	−5.7‰ at 25 °C but variable with temperature (Farquhar, 1983; Henderson *et al.*, 1992; Kromdijk *et al.*, 2010)
*C*_*BS*_	CO_2_ concentration in the BS; $C{\;}_{BS\;}=\frac{\frac{xJ_{ATP}}{2}-\frac{R_{LIGHT}}{2}-A}{g_{BS}}+C_{M}$	μmol·mol^−1^
*C*_*i*_	CO_2_ concentration in the intercellular spaces as calculated by the IRGA.	μmol·mol^−1^ (Li-cor 6400 manual eqn 1.18)
*C*_*M*_	CO_2_ concentration in the mesophyll; $C{\;}_{M\;}=C{\;}_{i\;}-\;\frac{A\;}{g_{M}}$	μmol·mol^−1^
*e*	^13^C fractionation during decarboxylation	0 to −10‰ (Gillon and Griffiths, 1997; Ghashghaie *et al.*, 2001; Igamberdiev *et al.*, 2004; Hymus *et al.*, 2005; Barbour *et al.*, 2007; Sun *et al.*, 2012); −6‰ in this study (Kromdijk *et al.*, 2010)
*e*′	^13^C fractionation during decarboxylation, including the correction for measurement artefacts: e′=e+δC13measurements−δC13growth chamber although there may be some error at low light intensities if recent photosynthate is not the substrate for respiration	‰ δ^13^C_measurements_ = −6.38‰; δ^13^C_growth chamber_ = −8‰ (Wingate *et al.*, 2007)
*e*_*s*_	^13^C fractionation during internal CO_2_ dissolution	1.1‰ (Vogel *et al.*, 1970; Mook *et al.*, 1974; Vogel, 1980)
*E*	transpiration rate (calculated by the IRGA software, parameter Trmmol)	mmol·m^−2^·s^−1^
*F*	Rate of photorespiratory CO_2_ evolution $F=0.5\cdot V_{O}\;$	μmol·m^−2^·s^−1^ (von Caemmerer, 2013; N. Ubierna, personal communication)
*f*	^13^C fractionation during photorespiration	11.6‰ (Lanigan *et al.*, 2008)
*GA*	Gross assimilation $GA=A\;+\;R_{LIGHT}$	μmol·m^−2^·s^−1^
*g*_*ac*_	conductance to diffusion of CO_2_ in air (calculated by the IRGA software, parameter CndCO2)	mol·m^−2^·s^−1^
*g*_*BS*_	BS conductance to CO_2_, calculated by fitting *J*_*MOD*_ to *J*_*ATP*_	mol·m^−2^·s^−1^ (Bellasio and Griffiths, 2013)
*g*_*M*_	Mesophyll conductance to CO_2_	1 mol·m^−2^·s^−1^·bar^−1^ (Kromdijk *et al.*, 2010)
*g*_*s*_	Stomatal conductance to CO_2_	mol·m^−2^·s^−1^
*J*_*ATP*_	ATP production rate $J{\;}_{ATP\;}=\frac{3\;GA{\;}_{Low\;O_{2}}\;Y(II)\;}{\;0.59\;Y(II){\;}_{Low\;O_{2}}}$	μmol·m^−2^·s^−1^ (Bellasio and Griffiths, 2013)
*J*_*ATP*_/*A*	ATP production rate relative to net assimilation	ATP / CO2
*J*_*ATP*_/*GA*	ATP production rate relative to gross assimilation	ATP / CO2
*J*_*MOD*_	Modelled ATP production rate $J{\;}_{MOD\;}=\frac{-\;y\;+\;\sqrt{y^{2}-4wz}}{2w}$ where: $w\;=\frac{x-x^{2}}{6A}$ $y\;=\frac{1-x}{3}\left[\frac{g_{BS}}{A}+\left(C_{M}-\frac{R_{M}}{g_{BS}}-\gamma^{\text{*}}O_{M}\right)-1-\frac{\alpha \gamma^{\text{*}}}{0.047}\right]-\frac{x}{2}\left(1+\frac{R_{LIGHT}}{A}\right)$ $z\;=\left(1+\frac{R_{LIGHT}}{A}\right)\left(R_{M}-g_{BS}C_{M}-\frac{7\;g_{BS}\gamma^{\text{*}}O_{M}}{3}\right)+\left(R_{LIGHT}+A\right)\left(1-\frac{7\alpha \gamma^{\text{*}}}{3\cdot 0.047}\right)$	μmol·m^−2^·s^−1^ (von Caemmerer, 2000; Bellasio and Griffiths, 2013; Ubierna *et al.*, 2013)
*O*_*BS*_	O_2_ mol fraction in the BS cells (in air at equilibrium) $O_{BS}=O_{M}+\;\frac{\alpha A}{0.047\;g_{BS}}$	μmol·mol^−1^ (von Caemmerer, 2000)
*O*_*M*_	O_2_ mol fraction in the mesophyll cells (in air at equilibrium)	210000 μmol·mol^−1^
*R*_*LIGHT*_	Respiration in the light	μmol·m^−2^·s^−1^
*R*_*M*_	Mesophyll non-photorespiratory CO_2_ production in the light R_M_ = 0.5 *R*_*LIGHT*_	μmol·m^−2^·s^−1^ (von Caemmerer, 2000; Kromdijk *et al.*, 2010; Ubierna *et al.*, 2013)
*s*	Fractionation during leakage of CO_2_ out of the BS cells	1.8‰ (Henderson *et al.*, 1992)
*t*	Ternary effects $t=\frac{\left(1+a\right)\;E}{2000\;g_{ac}}$	‰ (Farquhar and Cernusak, 2012)
*V*_*C*_	Rubisco carboxylation rate $V_{C}=\frac{\left(A+R_{LIGHT}\right)}{1-\frac{\gamma^{\text{*}}O_{BS}}{C_{BS}}}$	μmol·m^−2^·s^−1^ (Ubierna *et al.*, 2011)
*V*_*O*_	Rubisco oxygenation rate $V_{O}=\frac{V_{C}-A-R_{LIGHT}}{0.5}$	μmol·m^−2^·s^−1^ (Ubierna *et al.*, 2011)
*V*_*P*_	PEP carboxylation rate $V_{P}=\frac{xJ_{ATP}}{2}$	
*x*	*J*_*ATP*_ partitioning factor between C_4_ activity (*V*_*P*_) and C_3_ activity *V*_*C*_+*V*_*O*_ (reductive pentose phosphate pathway and photorespiratory cycle)	Set at 0.4 (von Caemmerer, 2000; Kromdijk *et al.*, 2010; Ubierna *et al.*, 2011, 2013), except for the calculation of eqn 2 where *x* was not constrained.
*Y(II)*	Yield of photosystem II $Y(II)=\frac{F_{m}^{\text{'}}-F_{s}}{F_{m}^{\text{'}}}$	dimensionless (Genty *et al.*, 1989)
α	Fraction of PSII active in BS cells	0.15 (von Caemmerer, 2000; Edwards and Baker, 1993; Kromdijk *et al.*, 2010)
γ*	Half of the reciprocal of the Rubisco specificity	0.000193 (von Caemmerer, 2000)
*Δ*	^13^C Isotopic discrimination $\Delta =\frac{\xi (\delta_{o}-\delta_{e})}{1+\delta_{o}-\xi (\delta_{o}-\delta_{e})}$ where: $\xi =\frac{C_{e}}{C_{e}-C_{o}}$ see supporting Fig. S1; δ_e_ is the isotopic composition of the reference gas. δ_o_ is the isotopic composition of the gas leaving the cuvette. *C*_*e*_ and *C*_*o*_ represent the CO_2_ mole fraction respectively entering and leaving the cuvette corrected for differing amounts of water vapour according to (von Caemmerer and Farquhar, 1981).	‰ (Evans *et al.*, 1986)
δ^13^C	^13^C isotopic composition relative to Pee Dee Belemnite	‰
*ϕ*	Leakiness; defined as the leak rate relative to *V*_*P*_ It was estimated with the isotope method including respiratory and photorespiratory fractionation, ternary effects and estimating C_BS_ with the C_4_ model $\phi \;=\;\frac{C{\;}_{BS\;}–C{\;}_{M\;}}{C{\;}_{M\;}}\frac{b{\;}_{4\;}C{\;}_{M\;}\left(1+t\right)+a\left(C{\;}_{a\;}–C{\;}_{i}\right)–C{\;}_{a\;}\Delta {\;}_{OBS\;}\left(1-t\right)}{\left(1+t\right)\left[C{\;}_{a\;}\Delta {\;}_{OBS\;}\left(1-t\right)–a\left(C{\;}_{a\;}–C{\;}_{i}\right)-b{\;}_{3\;}C{\;}_{BS\;}+s\left(C{\;}_{BS\;}–C{\;}_{M\;}\right)\right]}$	dimensionless (Farquhar and Cernusak, 2012)

The CCM has a notable metabolic cost: out of the theoretical minimum of five ATP molecules required for the *gross* assimilation of one CO_2_, two ATPs are consumed by the CCM (Furbank *et al.*, 1990; Bellasio and Griffiths, 2014) in the costly regeneration of PEP. The common interpretation of C_4_ physiology assumes that, at steady state, leaking CO_2_ is entirely refixed by PEP carboxylase (PEPC); hence, anatomical features are tightly bound to biochemical and energy traits. Plants with a higher *g*_*BS*_ would have higher rate of CO_2_ retrodiffusion, increased CCM cost, and a higher ATP demand for gross assimilation (ATP/*GA*), which is the overall biochemical operating efficiency of C_4_ photosynthesis. For this reason, *ϕ* has been used to derive ATP/*GA* (von Caemmerer, 2000; Tazoe *et al.*, 2008); for instance, plants with higher *ϕ* are considered to have higher ATP/*GA* and, therefore, lower biochemical operating efficiency. We will show that these assumptions hold true only under high light intensities.

Because of these anatomical, biochemical, and energetic complexities, C_4_ metabolism is highly sensitive to limiting light intensities (see Ubierna *et al.*, 2011 for review). Recently, studies have focused on characterizing the progressive increase in carbon isotope discrimination that is usually seen as light intensity decreases at both leaf (Tazoe *et al.*, 2008; Kromdijk *et al.*, 2010; Pengelly *et al.*, 2010; Bellasio and Griffiths, 2013; Ubierna *et al.*, 2013) and canopy (Kromdijk *et al.*, 2008) levels. The theoretical considerations highlighted above have associated this increase in *ϕ* with decreased C_4_ efficiency and a potential loss of photosynthetic carbon uptake (Furbank *et al.*, 1990; Kromdijk *et al.*, 2008; Tazoe *et al.*, 2008). Empirical evidence was needed to validate this suggestion and to explore the strategies that mature C_4_ leaves deploy to cope with reduced light intensities.

Low light responses are highly relevant for C_4_ canopy productivity, since up to 50% of net CO_2_ uptake (Baker *et al.*, 1988; Long, 1993) is fixed by shaded leaves, under a light intensity which is typically one-twentieth of full sunlight (Shirley, 1929). In a forest canopy leaves are subjected to a similar degree of exposure throughout the year, whereas in crop canopies most fully expanded leaves progressively acclimate to shade under newly emerging leaves. This long-term acclimation is accompanied by transitory, short-term responses such as daily shading, or more transient sunflecks. Furthermore, there is a gradient of leaf age down the crop canopy, with younger leaves exposed to full sunlight at the top of the canopy and older leaves subsequently exposed to canopy-filtered light.

Previously we studied how long-term acclimation to low light influenced short-term responses to illumination (Bellasio and Griffiths, 2013). Plants grown under low light (LL) showed a capacity for maintaining low *ϕ* even under decreasing light intensities, whereas *ϕ* increased in equivalent plants grown under high light (HL). We suggested several mechanisms whereby C_4_ leaves adapted *throughout* growth to low-light conditions could maintain high photosynthetic conversion efficiency during steady-state photosynthesis.

In this study we grew maize plants under a light regime representing the acclimation of leaves shaded by an over-growing canopy, consisting of 3 weeks under high light followed by 3 weeks under diffuse, low light intensity. The leaf-level ATP-production rate (*J*_*ATP*_) was derived from gas exchange measurements under low O_2_ in combination with photosystem II (PSII) photochemical yield, measured CO_2_ assimilation rate, and online isotopic discrimination during photosynthesis (*Δ*). A full isotopic discrimination model was used to derive *ϕ* from *Δ* (Farquhar, 1983; Ubierna *et al.*, 2011; Farquhar and Cernusak, 2012). With the directly derived values for *J*_*ATP*_, the empirical ATP cost of gross and net assimilation (respectively, *J*_*ATP*_/*GA* and *J*_*ATP*_/*A*) could be calculated and compared with the predicted ATP cost of assimilation (ATP/*GA*). Mature leaves that had re-acclimated under low light (HLLL) showed very similar traits to LL plants. HLLL plants deployed two strategies to optimize the scarce ATP resources under low light: (i) the reduction of respiration in the light (*R*_*LIGHT*_) and (ii) the reduction of leakiness (*ϕ*). The comparison of *J*_*ATP*_/*GA* with ATP/*GA* estimated with a novel metabolic model showed that C_4_ photosynthetic efficiency was constant in the vicinity of the light compensation point (LCP): thus, the predicted decrease in biochemical conversion efficiency based on *ϕ* increasing under limiting light does not occur.

## Materials and methods

### Plants

Plants were grown at the Plant Growth Facility located at the University of Cambridge Botanic Garden in controlled-environment growth rooms (Conviron, Winnipeg, Canada) set at 16-h day length, 25/23 °C (day/night), and 40% relative humidity. The growth protocol was designed to standardize age and watering conditions throughout the experiment. Two light environments were established—high-intensity direct light (photosynthetically active radiation, PAR = 600 μE·m^−2^·s^−1^) and low-intensity diffuse light (PAR = 100 μE·m^−2^·s^−1^)—obtained using shading to mimic the understory of a canopy. Maize seeds (*Zea mays* L. F_1_ hybrid PR31N27; Pioneer Hi-bred, Cremona, Italy) were sown weekly in 1.5 l pots filled with Levington pro M3 pot and bedding compost (Scotts, Godalming, Surrey, UK). Plants were grown in three sets of conditions: (i) HL plants were grown for 3 weeks under high light (fully expanded fourth leaf stage); (ii) LL plants were grown for 4 weeks under low light (fully expanded fourth leaf stage); and (iii) HLLL plants were grown for 3 weeks under high light, the youngest fully expanded leaf was marked, and then plants were grown for the following 3 weeks under low light. Plants were manually watered daily, with particular care to avoid overwatering. When ready, plants were measured once and then discarded. Measurements were performed on the youngest fully expanded leaf of HL and LL plants, and on marked leaves of HLLL plants.

### Gas exchange measurements with concurrent PSI/PSII yield and online carbon isotopic discrimination (*Δ*)

The experimental setup was previously described in detail (Bellasio and Griffiths, 2013). Briefly, an infrared gas analyser (IRGA; an LI6400XT, Li-cor, Lincoln, NE, USA) was fitted with a 6400–06 PAM2000 adapter and with a Li-cor 6400–18 red, green, and blue (RGB) light source. RGB light was used because, by providing equal fractions of R, G, and B, it is likely to distribute excitation between mesophyll and BS cells with a more similar pattern to natural white light than the conventional 90% R/10% B source. The IRGA was fed with CO_2_ (δ^13^C = −6‰; Isi Soda, Vienna, Austria) and either a mixture of 2% O_2_/N_2_ or ambient air. Photosystem I (PSI) yield and PSII yield (*Y(II)*; see Table 1) were measured using a Dual Pam-F (Heinz Walz GmbH, Effeltrich, Germany). Pulse intensity was set to 20 mE·m^−2^·s^−1^, enough to saturate fluorescence and PSI signals (which occurred between 8 and 10 mE·m^−2^·s^−1^; data not shown). The block temperature was set at 26 °C so as to maintain the leaf temperature close to 25 °C. The IRGA was connected to a cryogenic H_2_O- and CO_2_-trapping purification line. Each day, one plant was subject to a RGB-light-response curve, under 2% O_2_ and *C*_*a*_ = 600 μmol·mol^−1^ [to determine the relationship between electron transport rate (ETR) and *J*_*ATP*_] and a second RGB-light-response curve under 21% O_2_ and reference CO_2_ set at 400 μmol·mol^−1^, during which exhaust gas was trapped to determine *Δ*. With this procedure each day the δ^13^C composition of a total of 12 CO_2_ samples and six CO_2_ references (representing responses to decreasing irradiances of one individual plant) were analysed directly using a VG SIRA dual-inlet isotope ratio mass spectrometer (modified and maintained by Pro-Vac Services, Crewe, UK). *Δ* was calculated as reported in Table 1 (Evans *et al.*, 1986). *Y(II)* was determined at each light level for both light curves. *J*_*ATP*_ was calculated individually at each irradiance by multiplying the relationship between ETR and *J*_*ATP*_ (determined at low O_2_) by the ratio between *Y(II)* at ambient and low O_2_ (Table 1). *R*_*LIGHT*_ was calculated as the *y*-intercept of the linear regression of net assimilation, *A*, against $\mathrm{PAR}\;•\;\frac{Y(II)}{3}$ (Table 1; Yin *et al.*, 2011b; Bellasio and Griffiths, 2013). Although we did not find significant differences with values for dark respiration (measured with the IRGA every 10 s for 4min and averaged, with flow rate of 50 μmol·s^−1^) or with values of *R*_*LIGHT*_ estimated through non-linear curve fitting of light-response curves (non-rectangular hyperbola; Prioul and Chartier, 1977; Dougherty *et al.*, 1994), we preferred the linear curve fitting described above for the robustness and the simplicity detailed in Yin *et al.* (2011a). The LCP was calculated using dedicated software (Photosyn assistant 1.2, Dundee Scientific, Dundee, UK). Assimilatory light-response curves were transformed logarithmically and subject to analysis of variance (ANOVA; Genstat). The transformation was necessary to normalize the residuals and thereby avoid the artefactual intepretation of significance (i.e. significant differences only at higher light intensities). Responses to decreasing light intensities were subject to repeated-measures ANOVA (Genstat); point estimates were subject to ANOVA and Tukey multiple comparisons as appropriate (Genstat).

### Leakiness *ϕ* from isotopic discrimination *Δ*

Modelling was previously described in detail (Bellasio and Griffiths, 2013), and equations are reported in Table 1. Briefly, leakiness, *ϕ* was resolved from *Δ* using the full model of Farquhar, as recently integrated to take into account the ‘ternary’ effects, i.e. the effect of water molecules diffusing outward stomata on CO_2_ molecules diffusing inwards through still air (Farquhar and Cernusak, 2012). In this model, the weighted individual fractionations of the discriminating processes operating in C_4_ photosynthesis are summed. This model requires the CO_2_ concentration in the different cellular compartments (notably mesophyll and BS cells), which were calculated by means of the validated C_4_ photosynthesis model, in the light-limited form (later ‘C_4_ model’; von Caemmerer, 2000). The C_4_ model was in turn parameterized with the light-response data (*A*, *C*_*i*_, *C*_*a*_, *J*_*ATP*_) and *R*_*LIGHT*_. BS conductance, required to parameterize the C_4_ model, cannot be measured directly but it can be estimated by fitting the C_4_ model to a measured quantity. In the ‘Δ/Δ’ fitting (Kromdijk *et al.*, 2010; Ubierna *et al.*, 2013), the C_4_ model is rearranged to express a modelled isotopic discrimination and fitted to values for *Δ*. Here, we used the ‘J/J’ fitting, which we have recently described (Bellasio and Griffiths, 2013), whereby the C_4_ model is rearranged to express a modelled ATP production rate *J*_*MOD*_ and fitted to the empirically derived estimate for the leaf-level ATP-production rate *J*_*ATP*_, described above. This procedure yielded a value for *g*_*BS*_ for each individual plant which was obtained independently from *Δ*, and did not suffer the circularity of the ‘Δ/Δ’ fitting, arising from calculating *g*_*BS*_ and leakiness from the same values for *Δ* (Bellasio and Griffiths, 2013).

### Empirical and predicted ATP cost of gross assimilation

We refer to empirical ATP cost of net and gross assimilation as *J*_*ATP*_/*A* and *J*_*ATP*_/*GA*, while we refer to predicted ATP cost of *gross* assimilation as ATP/*GA*.

The empirical ATP cost of net and gross assimilation was calculated from the data obtained during the experiment. Firstly, the measured leaf-level ATP cost of *net* assimilation (*J*_*ATP*_/*A*) was calculated from *J*_*ATP*_ and net assimilation, *A*. The derivation of *J*_*ATP*_ through the low O_2_-ETR method was described above (see also Table 1). *J*_*ATP*_/*A* is relevant to net productivity and shows how much ATP the plant has to spend for net gain of a CO_2_ molecule. Then, the leaf-level ATP cost of *gross* assimilation (*J*_*ATP*_/*GA*) was calculated using values for *GA*, derived by summing *A* plus *R*_*LIGHT*_ calculated by curve fitting (see above). *J*_*ATP*_/*GA* is relevant to C_4_ biochemistry and shows the empirical conversion efficiency of CO_2_ into sugars. It is worth stressing that these values for *J*_*ATP*_/*GA* are derived with a novel method based on gas exchange under low O_2_ (Yin *et al.*, 2011a, 2011b; Bellasio and Griffiths, 2013). The low O_2_-ETR method relies on two assumptions. First is that the partitioning of NADPH to photosynthesis does not change between ambient and low O_2_. This is a fair assumption since NADPH use by alternative sinks (e.g. nitrogen reduction) is generally dependent on light intensity and hence it is only marginally influenced by O_2_ partial pressure (Yin *et al.*, 2004, 2009; Yin and Struik, 2012). Second, *R*_*LIGHT*_ does not vary between low and ambient O_2_. This is also a fair assumption because any O_2_ effect is generally negligible (Badger, 1985; Gupta *et al.*, 2009). The low O_2_-ETR method does not rely on the assumptions used in the traditional derivation based on leaf absorptance and PSII optical section (von Caemmerer, 2000) and should therefore better represent the actual biochemical ATP demand of the portion of leaf subject to ecophysiological characterization. Because of the difficulty in deriving *J*_*ATP*_/*GA* based on leaf absorptance, and the difficulty in capturing the stoichiometry at the electron transport chain, the ATP cost of gross assimilation has often been predicted (e.g. Tazoe *et al.*, 2008).

A traditional way to predict ATP/*GA* uses leakiness as the sole proxy (*ϕ* approach; Furbank *et al.*, 1990; von Caemmerer, 2000; Tazoe *et al.*, 2008). The *ϕ* approach relies on the assumption that the ATP cost of the C_3_ activity is invariably 3 ATP/CO_2_ (photorespiration is neglected) while the ATP cost of the CCM depends solely on *ϕ*. This implies that the CCM is driven solely by the activity of PEPC and that all the retrodiffusing CO_2_ is refixed. Under these assumptions the ATP cost of the CCM is calculated by multiplying the overall ATP cost of PEPC (2 ATP/CO_2_) by the ratio of CO_2_ overcycling [1/(1−*ϕ*)]. The total ATP cost of gross assimilation results from summing the cost of the C_3_ activity plus the cost of the CCM (see eqn 5 in Tazoe *et al.* 2008, or eqn 4.55 in von Caemmerer, 2000):

$${\frac{ATP}{GA}}_{\Phi }=3+\frac{2}{1\;\;-\;\Phi }$$ (1)

Here the subscript *ϕ* recalls that ATP/*GA* is derived from leakiness. Eqn 1 was solved for the three types of plants (HL, LL, and HLLL) and light intensities from 50 to 500 μE·m^−2^·s^−1^ using the values of *ϕ* derived from isotopic discrimination.

We propose a different approach to estimate ATP/*GA*, whereby the ATP demand of all biochemical processes underpinning assimilation (hence B approach) are summed. The B approach is comprehensive, and requires the quantification of all processes contributing to C_4_ photosynthesis. We used the validated C_4_ model (von Caemmerer, 2000), as recently integrated to describe the C_4_ energetics (Bellasio and Griffiths, 2014). The biochemical processes considered are: 3-phosphoglyceric acid (PGA) reduction, starch synthesis, PEP regeneration, ribulose 1,5-bisphosphate (RuBP) regeneration, and glycolate recycling, while the PGA consumed by mitochondrial respiration is subtracted as likely to be consumed by basal metabolism (for derivation see Bellasio and Griffiths, 2014). ATP/*GA*_*B*_ was calculated as:

$${\frac{ATP}{GA}}_{\text{B}}=3V_{C}+\frac{7}{2}V_{O}+\frac{A}{6}+PEPCK+2PPDK-\frac{1}{3}R_{LIGHT}$$ (2)

**Fig. 1.**
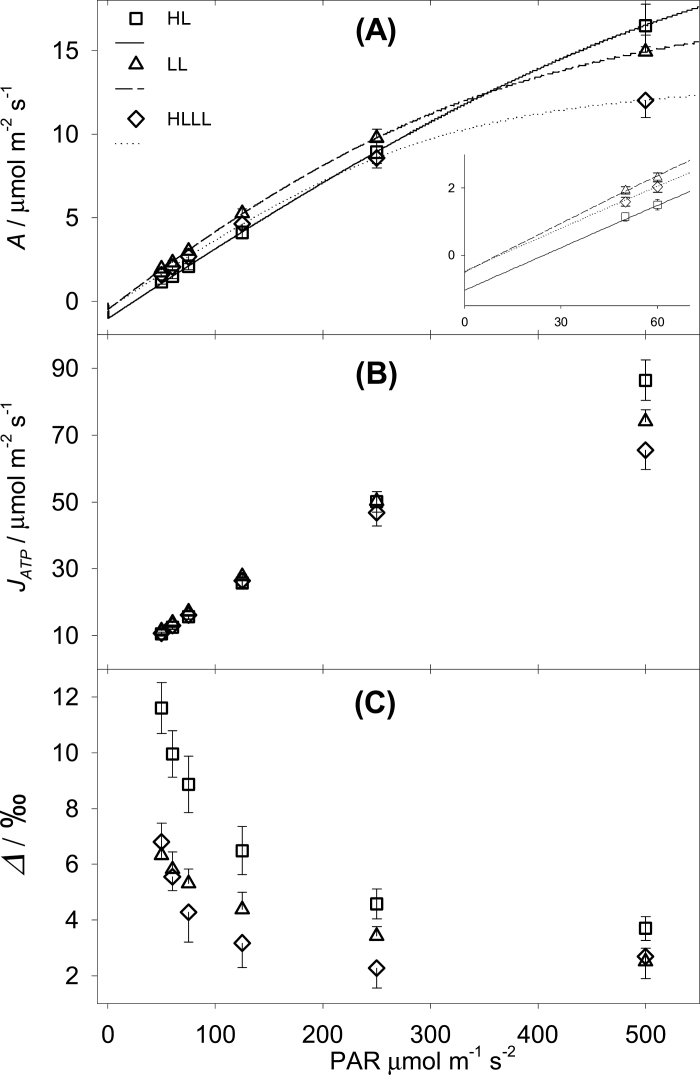
Maize responses to decreasing light intensities for plants grown under high light (HL), low light (LL) or LL following HL (HLLL). (A) Net assimilation (*A*). The curves were fitted to calculate the LCP (Table 2). The inset shows a magnification at the lowest PAR. (B) Total ATP production rate (*J*_*ATP*_), measured with the low O_2_-ETR method (see Materials and methods section on gas exchange measurements). (C) Online isotopic discrimination during photosynthesis (*Δ*). Error bars represent one SE (*n* = 6).

**Fig. 2.**
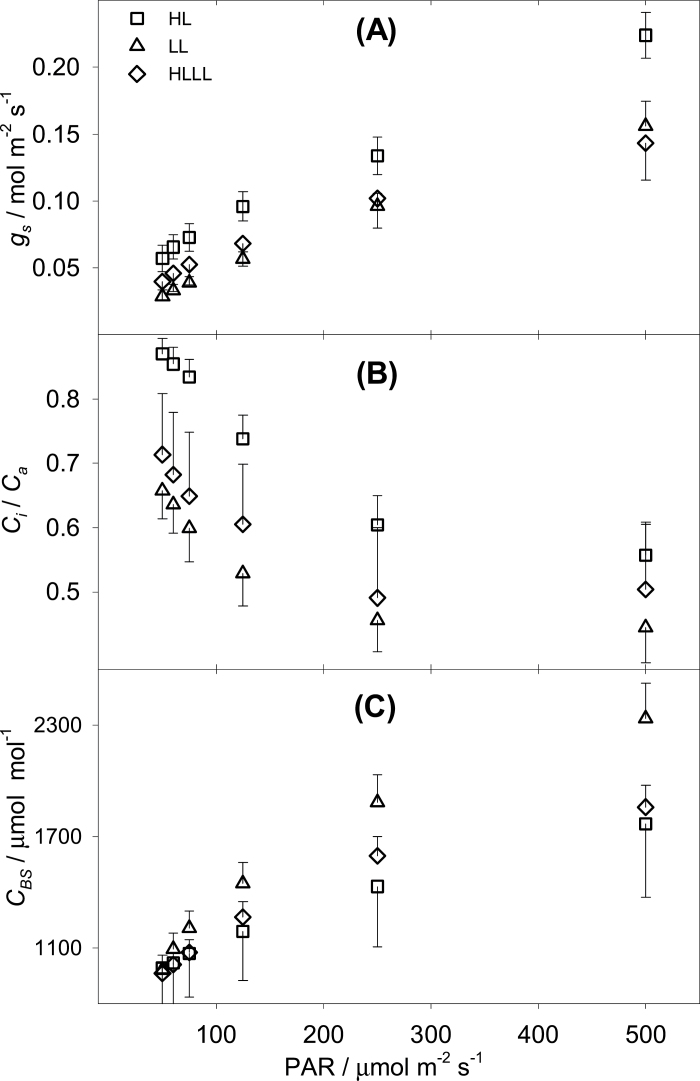
(A) Stomatal conductance and (B) *C*_*i*_/*C*_*a*_ responses to decreasing light intensity, under different light qualities, for plants grown under high light (HL), low light (LL), or LL following HL (HLLL) measured by gas exchange. (C) Response of *C*_*BS*_ to decreasing light intensity, under different light qualities, estimated by the C_4_ model. Error bars represent one SE (*n* = 6).

**Fig. 3.**
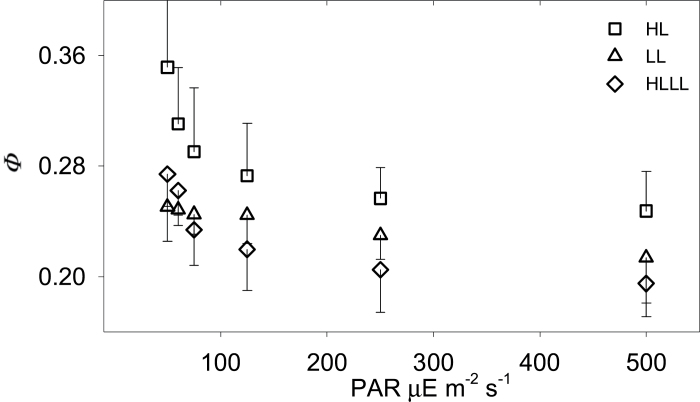
Leakiness (*ϕ*) resolved from online isotopic discrimination during photosynthesis (*Δ*) by means of a full isotopic discrimination model for HL plants (squares), LL plants (triangles), and HLLL plants (diamonds). Error bars represent one SE (*n* = 6).

Where the subscript *B* recalls that all the biochemical processes were summed, *V*_*C*_ is the Rubisco carboxylation rate, *V*_*O*_ is the Rubisco oxygenation rate, *A* is net assimilation, *PEPCK* is the PEP carboxykinase (PEPCK) rate, and *PPDK* is the pyruvate phosphate dikinase (PPDK) rate. PEPCK was assumed to regenerate 20% of the PEP required by PEPC, which is close to the expected value under natural white light (Bellasio and Griffiths, 2014); the remainder was regenerated through PPDK. PEPC rate (*V*_*P*_), *V*_*C*_, and *V*_*O*_ were calculated with the validated von Caemmerer C_4_ model (Table 1), in the light-limited form (von Caemmerer, 2000; Bellasio and Griffiths, 2013). The model was constrained at each light intensity with the values for *A* and *J*_*ATP*_ shown in Fig. 1, with the values for *C*_*i*_/*C*_*a*_ and *C*_*BS*_ shown in Fig. 2, with the values for *R*_*LIGHT*_ and *g*_*BS*_ reported in Table 2, and with the values for *ϕ* shown in Fig. 3. A parameter, known as *x*, is required to solve the C_4_ model (Table 1), which partitions the ATP available between the CCM activity and the C_3_ activity (PGA reduction, RuBP regeneration, and glycolate recycling). For the purposes of these calculations, rather than using a fixed value of *x*, such as 0.4 (von Caemmerer, 2000; Kromdijk *et al.*, 2010), we allowed *x* to vary to get the best fit for the parameters above.

**Table 2.** Physiological responses for plants grown under high light (HL), low light (LL) or LL following HL (HLLL) The LCP was determined by fitting light curves with dedicated software; *R*_LIGHT_ was determined by linear regression of *A* against PAR∙*Y(II)*/3; BS conductance (*g*_BS_) was determined by fitting a modelled *J*_MOD_ to the measured *J*_ATP_ (Fig. 3). Different letters identify significant differences across rows at *P* < 0.05 in a Tukey multiple comparison test (Genstat). Mean values ± SE are shown; *n* = 6 per treatment.

	Unit	Mean	HL	LL	HLLL
LCP	μE·m^−2^·s^−1^	15.3	24.4±1.9^a^	10.4±0.65^b^	11.2±1.0^b^
*R*_*LIGHT*_	μmol·m^−2^·s^−1^	0.680	1.05±0.14^a^	0.510±0.057^b^	0.477±0.053^b^
*g*_*BS*_	mol·m^−2^·s^−1^	0.000944	0.00136±5.2×10^−4 a^	0.000647±9.2×10^−5 a^	0.000822±1.9×10^−4 a^

## Results

### Physiological response to decreasing light intensities

Figure 1 shows the responses of maize plants to decreasing irradiance when grown under three different light regimes. Assimilation (*A*) significantly differentiated plant responses (Fig. 1A). LL plants had the highest *A* at PAR lower than 500 μE·m^−2^·s^−1^. HL plants had the highest *A* at saturating PAR and the lowest *A* at PAR lower than 250 μE·m^−2^·s^−1^. HLLL plants had the lowest *A* under saturating light (although these leaves were now 3 weeks older), but as light decreased between PAR 250 and 0 μE·m^−2^·s^−1^ the response approached that of LL plants. Consistently, the LCP and *R*_*LIGHT*_ of HLLL plants were similar to those of LL plants, and clearly lower than those of HL plants (Table 2).

The total ATP production rate (*J*_*ATP*_) is shown in Fig. 1B. *J*_*ATP*_ was derived from gross assimilation under low O_2_ and then corrected for photorespiration at ambient O_2_ using the ratio of photochemical yield. At high PAR, *J*_*ATP*_ tracks the pattern of *A*; however, at low PAR, *J*_*ATP*_ of all plants was similar, suggesting that the higher *A* of LL and HLLL plants at limiting PAR (inset in Fig. 1A) was achieved through a higher conversion efficiency and lower respiration rate (Table 2). Isotopic discrimination during photosynthesis (*Δ*) is shown in Fig. 1C. In HL and HLLL plants *Δ* increased substantially at PAR lower than 250 μE·m^−2^·s^−1^, although in HLLL plants *Δ* was, on average, lower than for HL plants. LL plants showed a more gradual increase under decreasing PAR.

Figure 2A shows stomatal conductance (*g*_*s*_) and Fig. 2B shows *C*_*i*_/*C*_*a*_. *C*_*i*_/*C*_*a*_ differentiated clearly between growth conditions, and was lowest in LL plants and highest in HL plants, while HLLL plants had intermediate values at all levels of PAR. *C*_*i*_/*C*_*a*_ was higher than 0.5 at PAR <125 μE·m^−2^·s^−1^ (LL plants), reflecting the efforts made during the measurements to induce stomatal opening. A high *C*_*i*_/*C*_*a*_ was functional in the resolution of the isotopic discrimination model, to maximize the contribution of biochemical processes over the stomatal contribution to total isotopic discrimination (Table 1; Cernusak *et al.*, 2013). This was especially important for HL plants which have, under low light, lower assimilation than LL plants (and higher ξ, Table 1, Fig. S1; Evans *et al.*, 1986). Figure 2C shows the CO_2_ concentration in BS (*C*_*BS*_), which was estimated by fitting a C_4_ photosynthesis model, rearranged to express *J*_*MOD*_, to the values for *J*_*ATP*_ described above. The difference between conditions was not significant, and was due to a small difference in permeability to CO_2_ retrodiffusion out of the BS (*g*_*BS*_; Table 2).

### Leakiness

Figure 3 shows leakiness, *ϕ*, over the experimental range of PAR. These values were resolved from *Δ* through a full isotopic discrimination model (Farquhar and Cernusak, 2012), parameterized using the C_4_ model and fitted to *J*_*ATP*_, using the recently described J/J fitting (Bellasio and Griffiths, 2013). *ϕ* significantly differentiated the three types of plants (*P* = 0.008). HL plants had higher *ϕ* than LL and HLLL under limiting PAR, with *ϕ* increasing from 0.25 to 0.35 under decreasing PAR. HLLL plants had the lowest *ϕ* under light intensities higher than 75 μE·m^−2^·s^−1^. Under low light intensities they showed an increase in *ϕ* with a similar trend to that of HL plants. In LL plants *ϕ* was close to 0.24 and only marginally affected by light intensity.

### ATP cost of assimilation

**Fig. 4.**
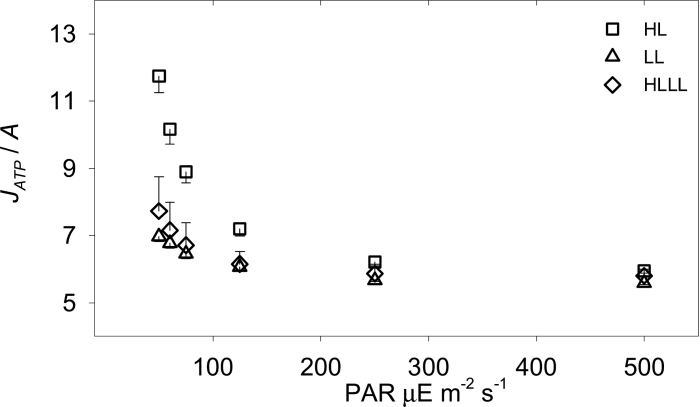
Measured ATP cost of net assimilation (*J*_*ATP*_/*A*) for HL plants (squares), LL plants (triangles), and HLLL plants (diamonds). Error bars represent one SE (*n* = 6).

**Fig. 5.**
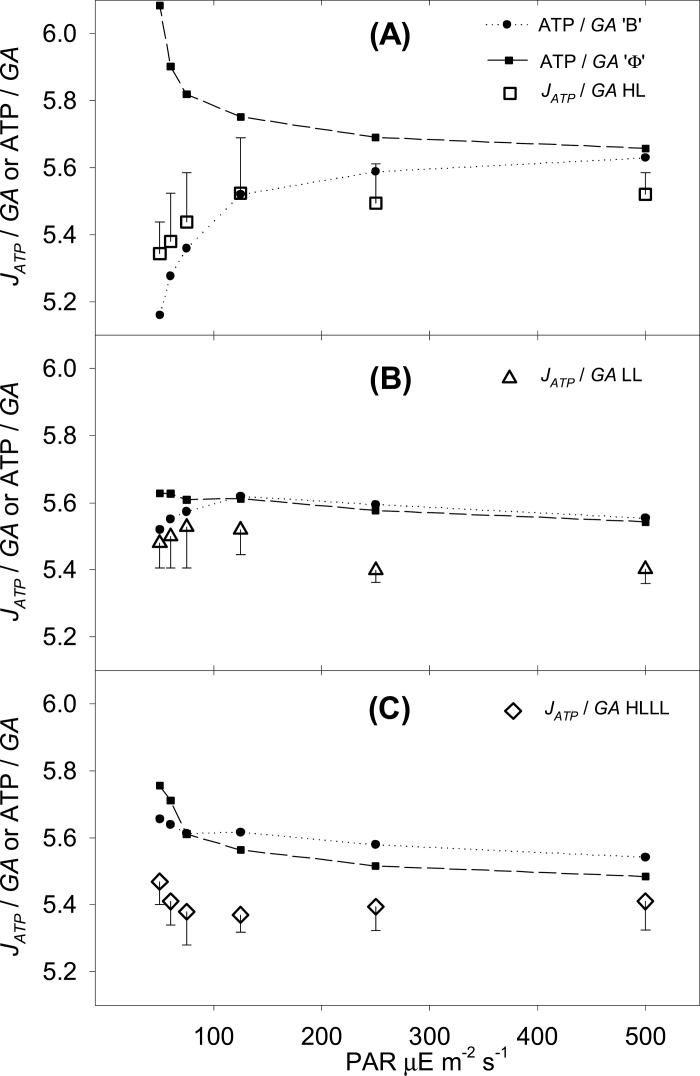
ATP cost of gross assimilation, representing C_4_ biochemical operating efficiency for HL plants (A), LL plants (B), and HLLL plants (C). The empirical values for *J*_*ATP*_/*GA* (empty symbols) were compared to predicted values for ATP/*GA* (solid symbols) calculated with two different approaches. ATP/*GA* was calculated using *ϕ* as the sole proxy for operating efficiency (*ϕ* approach; solid squares) or using a comprehensive calculation summing the ATP cost of all processes contributing to assimilation (B approach; solid circles). Note that both calculations were based on the same dataset, presented in Figs 1–3 and Table 2. Error bars represent one SE; *n* = 6 plants per condition.

Two empirical ATP costs of (net and gross) assimilation were derived. Figure 4 shows the empirical ATP cost of net assimilation *J*_*ATP*_/*A*. This quantity expresses the ATP cost involved in the assimilation of CO_2_; that is, how much ATP the plant has to produce (= consume, at steady state) to assimilate one CO_2_ molecule. Figure 4 shows clearly that *J*_*ATP*_/*A* for HLLL plants was very similar to that of LL plants and significantly lower than that of HL plants. This means that re-acclimation was extremely effective in reducing *J*_*ATP*_/*A*, particularly in the vicinity of the LCP. Figure 5 shows the ATP cost of gross assimilation, *J*_*ATP*_/*GA*. This quantity is the biochemical conversion efficiency of C_4_ assimilation, or how much ATP is needed to convert bicarbonate into stable assimilates. The empirical values for *J*_*ATP*_/*GA* (Fig. 5, symbols in panels A–C) were close to 5.4 and not significantly influenced by light intensity or by the growth light regime. This means that, in contrast to the common interpretation, the biochemical conversion efficiency was not affected by instantaneous light intensity.

To support this result theoretically, we predicted ATP/*GA* with two different approaches. These methods are compared in Fig. 5. A simplified method used *ϕ* as a sole proxy for C_4_ operating efficiency (*ϕ* approach; Fig. 5, solid squares), whereas the complete biochemical method (B approach; Fig. 5, solid circles) summed the individual ATP demands of processes involved in assimilation. Under low light intensities the *ϕ* approach resulted in an overestimation of *J*_*ATP*_/*GA*, especially in HL plants (Fig. 5A), which display the characteristic hyperbolic *ϕ* increase in proximity of the compensation point (Fig. 3). Under higher irradiances or when *ϕ* was lower, the *ϕ* approach resulted in an accurate estimation of *J*_*ATP*_/*GA*. The B approach provided a better estimate of *J*_*ATP*_/*GA*, across the range of incident light intensities and independent of the values for *ϕ.* It is worth stressing that both these estimates were based on the same dataset shown in Fig. 3, but, although the *ϕ* approach translated the *ϕ* pattern shown in Fig. 3 directly into ATP cost, the B approach considered the rates of the underpinning biochemical reactions and summed the ATP costs involved in each individual process.

## Discussion

Maize plants were grown under high light then re-acclimated to diffuse low irradiance, and compared to plants grown either under high light or low light. The particular conditions of re-acclimation were intended to represent the transition from full sunlight to shaded conditions that maize leaves undergo when overgrown by newly emerging leaves at the top of the canopy. This is a natural acclimation process for maize leaves, in fact for the experimental plants all leaves grown under HL were retained throughout the re-acclimation period, and continued to photosynthesize under low irradiance, although we cannot exclude the possibility that such acclimation to low light could also include changes in development or source/sink relations. The opposite acclimation is not likely to be a physiologically realistic process, and, in fact, when LL plants were moved to higher light intensities they promptly shed all leaves grown under low light. The natural re-acclimation to low light brought about substantial physiological changes, which have implications for the energy balance of leaves within a growing canopy.

### Acclimation strategies

The three types of plants were subject to concurrent gas exchange, variable fluorescence, and isotopic discrimination measurements. Direct estimates for *J*_*ATP*_ were derived from a combined low O_2_-ETR method, and leakiness was derived from isotopic discrimination. This comprehensive ecophysiological characterization highlighted two main re-acclimation strategies.

A first strategy involved reducing *R*_*LIGHT*_. This reduction is underpinned by considerable changes at the metabolic level that result in reducing the level of basal metabolism. This had a direct effect on the LCP and, for this reason, it had a direct effect on the ATP cost of net assimilation (see below). A second strategy involved the reduction of leakiness (*ϕ*). HLLL plants showed reduced values for *ϕ* as compared to HL plants. However the *ϕ* hyperbolic increase under low irradiance was similar to that of HL plants. In contrast, and in agreement with recent results (Bellasio and Griffiths, 2013; Ubierna *et al.*, 2013), LL plants showed a linear trend, with *ϕ* values that were only marginally affected by irradiance. Further model outputs suggested that the general reduction in *ϕ* observed for HLLL plants (Fig. 3) could only be partially accounted by changes in *g*_*BS*_ (Table 2), and it may therefore be regulated at the level of relative rates of *V*_*P*_ and *V*_*C*_, as we have discussed previously (Bellasio and Griffiths, 2013). The observed plasticity in *ϕ* may then involve tuning of biochemical reaction rates and, in particular, the ratio between the CCM activity and the C_3_ activity, or the capacity to accommodate *g*_*BS*_ in response to light intensity, as we have recently hypothesized (Bellasio and Griffiths, 2013). However, the ultimate nature of this tuning is still speculative.

Overall, these strategies were highly effective in reducing the ATP cost of net assimilation *J*_*ATP*_/*A*. In fact, under a PAR of 50 μE·m^−2^·s^−1^ *J*_*ATP*_/*A* for HLLL plants was 35% lower than that of HL plants and very similar to that of LL plants (Fig. 4). However, this ATP cost reduction was largely associated with the reduced *R*_*LIGHT*_. In fact, when the effect of *R*_*LIGHT*_ reduction was isolated and the biochemical operating efficiency (i.e. the ATP cost of gross assimilation *J*_*ATP*_/*GA*) was considered, only minor energetic differences could be observed between different light treatments. Re-acclimation significantly influenced neither the empirical *J*_*ATP*_/*GA* (mean of 5.47 for HLLL and 5.45 for HL, as compared to 5.40 for LL) nor the predicted ATP cost of GA (ATP/*GA*, B approach; mean 5.56 for HLLL and 5.42 for HL, as compared to 5.61 for LL). This shows that if there were any effect of varied *ϕ* on the overall biochemical conversion efficiency the effect was undetectable using the methods described. On one hand this confirms the difficulties in estimating leakiness from leaf-level energetics (Furbank *et al.*, 1990; Kromdijk, 2010), on the other it highlights the complexity of the leakiness phenomenon, which depends at the same time on anatomical and biochemical traits. In this study we have specifically addressed the ATP demand, but other aspects are intertwined and may all contribute to *ϕ* dynamics. These could include (von Caemmerer and Furbank, 2003; Furbank, 2011; Bellasio and Griffiths, 2014) (i) regulating the ratio of C_4_ dicarboxylic acid to amino acid export to BS, (ii) regulating reducing power export from mesophyll to BS cells in response to demand, (iii) partitioning metabolic work between contrasting cells types (e.g. PGA reduction, starch synthesis, glycolate recycling, RuBP + PEP regeneration), (iv) optimizing energy availability in BS and mesophyll cells while at the same time (v) maintaining the equilibrium between the CCM and the C_3_ activity, and, finally, (vi) trading-off at the level of BS conductance, between the capacity to support very high diffusion (and assimilation) rates and the necessity to limit leakage of CO_2_ out of the BS (Sowinski *et al.*, 2008).

### Predicting C_4_ operating efficiency

The ‘conventional’ approach to predicting C_4_ biochemical operating efficiency (i.e. the ATP cost of gross assimilation, ATP/*GA*) uses leakiness, *ϕ* as the sole proxy (eqn 2, referred as the *ϕ* approach). With the *ϕ* approach the C_3_ activity is considered to have an invariable cost of 3 ATP/*GA* (photorespiration is neglected), whereas the CCM is assumed to be supplied solely by PEPC activity, which is assumed to refix entirely the retrodiffused (leaked) CO_2_. Our empirical evidence largely confirms the validity of the *ϕ* approach, which closely predicted the trend and the magnitude of *J*_*ATP*_/*GA* under PAR >125 μE·m^−2^·s^−1^ for LL + HLLL plants, and under PAR ≥500 μE·m^−2^·s^−1^ for HL plants. However, under low irradiances, the *ϕ* approach overestimated the trend of *J*_*ATP*_/*GA*, especially for HL plants. This overestimation is dependent on the assumptions of the *ϕ* approach (photorespiration is neglected and the CCM is driven solely by PEPC), which hold only under high irradiances, while under low irradiance they are no longer valid. In fact, PEPC and Rubisco activities proportionally decrease under decreasing irradiance, limited by the decreasing ATP availability. As opposed to that, BS respiration is largely unaffected by light intensity, and, under decreasing light intensities, the BS-respired CO_2_ progressively outweighs PEP carboxylation rate (*V*_*P*_). Hence, *ϕ*—that is, the ratio of retrodiffusing CO_2_ over PEP carboxylation rate—becomes progressively higher as light intensity approaches the compensation point. This gives rise to the extensively documented (empirically and theoretically) hyperbolic *ϕ* increase (for review see Ubierna *et al.*, 2011), which can be largely supplied by respiration without an additional engagement of PEPC. A constant degree of engagement of PEPC, even under the hyperbolic *ϕ* increase, is consistent with the observation that both the ratio of PEPC/Rubisco carboxylation rate (*V*_*P*_/*V*_*C*_) and the optimal partitioning factor between the CCM activity and the C_3_ activity (*x*) are largely independent of light intensity (von Caemmerer, 2000; Kromdijk *et al.*, 2010).

These considerations can be better appreciated if the CCM is viewed as complex machinery. The activity of PEPC is only one of the systems which contribute to loading CO_2_ into the BS. Recently it has become increasingly clear how photorespiration may contribute to the CCM, and is the predominant driving force in evolutionally early types of CCM (Sage *et al.*, 2012; Schulze *et al.*, 2013). We have shown in this paper and in previous work (Bellasio and Griffiths, 2013) that the CCM can be increasingly supplied by respiration under limiting light conditions, bringing about increased leakiness even without a predicted increase in the activity of PEPC. It is worth remembering that the compartmentalization of photochemical water oxidation to mesophyll cells, whose degree may vary considerably between subtypes and along the evolutionary line (Meierhoff and Westhoff, 1993; Sage, 2004; Furbank, 2011; Sage *et al.*, 2011), also contributes to increasing the ratio of O_2_/CO_2_ at the active site of Rubisco and should also be considered as a component of the complex machinery of the CCM.

In view of this complexity, leakiness, which reflects inherently complex biochemical and anatomical traits, should only be used to predict the magnitude (and ATP cost) of the CCM under high light regimes. However, we showed that *J*_*ATP*_/*GA* could be closely predicted using a complete biochemical approach (B approach, eqn 2), whereby the ATP cost of all processes contributing to assimilation are summed. We used the equations that we have recently derived (Bellasio and Griffiths, 2014), which are based on the comprehensive description of the C_4_ metabolism outlined by Furbank (2011) and on the validated C_4_ model (von Caemmerer, 2000, 2013). Within this approach, the ATP-consuming processes considered are PGA reduction, PEP regeneration (through PPDK and PEPCK), and starch synthesis. Furthermore the B approach subtracts the PGA used by respiration, which does not need to be reduced (PGA reduction to DHAP consumes 1 ATP and 1 NADPH). With this comprehensive calculation, the empirical data could be closely predicted in the vicinity of the LCP. Notably, under decreasing light intensities, the biochemical conversion efficiency did not decrease, regardless of the hyperbolic *ϕ* increase observed in HL and HLLL plants.

## Conclusion

In this study we set out to investigate the strategies deployed by maize plants grown under high light intensities when re-acclimated to low light. We showed that the main re-acclimation drivers were the reduction of respiration and the reduction of leakiness, and these were likely to be accompanied by complex metabolic reorganization. Overall, these strategies were very effective in reducing the ATP cost of *net* assimilation under low light intensities, which, for HLLL plants, decreased by 35% as compared to HL plants under PAR = 50 μE·m^−2^·s^−1^. This shows clearly that the net energy conversion efficiency under limiting light is to a considerable extent ameliorated by the acclimation of mature leaves to low light.

By calculating ATP cost of *gross* assimilation we could isolate the contribution of day respiration from the other biochemical effects (which include the reduction of leakiness). The ATP cost of gross assimilation was not significantly different for HLLL plants as compared to HL plants. This showed that re-acclimation did not change the efficiency of C_4_ metabolism, even if it considerably reduced leakiness, implying that the effect of reduced leakiness on C_4_ energetics was not detectable. Leakiness dynamics may then be associated to other processes occurring at biochemical level such as the regulation between BS versus mesophyll metabolic engagement and CCM versus C_3_ activity. In addition, we provided compelling theoretical and empirical evidence showing that the increase in hyperbolic leakiness, observed under low light intensities (Fig. 3), is not associated with a loss of energetic efficiency. The well-consolidated idea of C_4_ efficiency loss under low light conditions (e.g. von Caemmerer, 2000; Tazoe *et al.*, 2008) relies on assumptions that should be reconsidered in view of recent discoveries: the CCM is not uniquely supplied by the ATP-costly PEPC activity, but, under certain conditions, the contribution through respiration and photorespiration may be significant (Kromdijk *et al.*, 2010; Sage *et al.*, 2012; Bellasio and Griffiths, 2013). We proposed a comprehensive biochemical method (Bellasio and Griffiths, 2014) based on the validated C_4_ model (von Caemmerer, 2000). The biochemical method predicts the C_4_ conversion efficiency (as ATP cost of gross assimilation), taking into account the active and passive contributions to the CCM.

The implications for loss of productivity at the field scale being specifically associated with increased leakiness (Kromdijk *et al.*, 2008) may be less severe than previously thought. However, here we have shown the potential for acclimation with a somewhat extreme acclimation pattern whereby mature leaves were switched from the full light to deep shade. Realistically, mature leaves will undergo a more gradual transition from full sunlight through a condition characterized by rapid changes in irradiance (daily shading, sunflecks), to complete shade. The actual extent to which leaves optimize energy efficiency, when exposed to such a complex pattern of illumination under field conditions, remains to be addressed.

## Supplementary material

Supplementary material is available at *JXB* online.

Supplementary Fig. S1. ξ values for the calculation of *Δ* (equations are reported in Table 1).

Supplementary Data

## Abbreviations:

BS: bundle sheath
CCM: carbon-concentrating mechanism
ETR: electron transport rate
HL: plants grown under high light
HLLL: mature leaves re-acclimated under low light
IRGA: infrared gas analyser
LCP: light compensation point
LL: plants grown under low light
PAR: photosynthetically active radiation
PEP: phosphoenolpyruvate
PEPC: phosphoenolpyruvate carboxylase
PEPCK: phosphoenolpyruvate carboxykinase
PGA: 3-phosphoglyceric acid
PPDK: pyruvate phosphate dikinase
PSI: photosystem I
PSII: photosystem II
RGB: red, green, and blue
RuBP: ribulose 1,5-bisphosphate.
