# Supplementary material for: Acclimation of C4 metabolism to low light in mature maize leaves could limit energetic losses during progressive shading in a crop canopy
Source: J Exp Bot. 2014 Mar 3;65(13):3725–36. doi: 10.1093/jxb/eru052 (PMC4085954; doi:10.1093/jxb/eru052)
Supplement: Supplementary Data [file supp_65_13_3725__index.html]

Acclimation of C4 metabolism to low light in mature maize leaves could limit energetic losses during progressive shading in a crop canopy — Acclimation of C4 metabolism to low light in mature maize leaves could limit energetic losses during progressive shading in a crop canopy — Supplementary Data 

# Acclimation of C4 metabolism to low light in mature maize leaves could limit energetic losses during progressive shading in a crop canopy

## Supplementary Data

Data files

**Files in this Data Supplement:**

- Supplementary Data - Supplementary Data
