# Supplementary material for: Acclimation of C4 metabolism to low light in mature maize leaves could limit energetic losses during progressive shading in a crop canopy
Source: J Exp Bot. 2014 Mar 3;65(13):3725–36. doi: 10.1093/jxb/eru052 (PMC4085954; doi:10.1093/jxb/eru052)

Supplementary Figure S 1. *ξ* values for the calculation of *Δ* (Table 1). Error bars show one standard error. n=6.

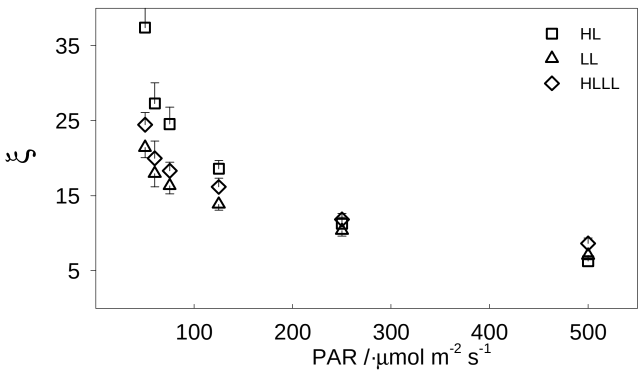

Supplement: Supplementary Data [file supp_eru052_jexbot113985_file001.pdf]
